# Anti-CD25 Treatment Depletes Treg Cells and Decreases Disease Severity in Susceptible and Resistant Mice Infected with *Paracoccidioides brasiliensis*

**DOI:** 10.1371/journal.pone.0051071

**Published:** 2012-11-30

**Authors:** Maíra Felonato, Adriana Pina, Eliseu Frank de Araujo, Flávio V. Loures, Silvia B. Bazan, Cláudia Feriotti, Vera L. G. Calich

**Affiliations:** Departamento de Imunologia, Instituto de Ciências Biomédicas, Universidade de São Paulo, São Paulo, São Paulo, Brazil; New York University, United States of America

## Abstract

Regulatory T (Treg) cells are fundamental in the control of immunity and excessive tissue pathology. In paracoccidioidomycosis, an endemic mycosis of Latin America, the immunoregulatory mechanisms that control the progressive and regressive forms of this infection are poorly known. Due to its modulatory activity on Treg cells, we investigated the effects of anti-CD25 treatment over the course of pulmonary infection in resistant (A/J) and susceptible (B10.A) mice infected with *Paracoccidioides brasiliensis*. We verified that the resistant A/J mice developed higher numbers and more potent Treg cells than susceptible B10.A mice. Compared to B10.A cells, the CD4^+^CD25^+^Foxp3^+^ Treg cells of A/J mice expressed higher levels of CD25, CTLA4, GITR, Foxp3, LAP and intracellular IL-10 and TGF-β. In both resistant and susceptible mice, anti-CD25 treatment decreased the CD4^+^CD25^+^Foxp3^+^ Treg cell number, impaired indoleamine 2,3-dioxygenase expression and resulted in decreased fungal loads in the lungs, liver and spleen. In A/J mice, anti-CD25 treatment led to an early increase in T cell immunity, demonstrated by the augmented influx of activated CD4^+^ and CD8^+^ T cells, macrophages and dendritic cells to the lungs. At a later phase, the mild infection was associated with decreased inflammatory reactions and increased Th1/Th2/Th17 cytokine production. In B10.A mice, anti-CD25 treatment did not alter the inflammatory reactions but increased the fungicidal mechanisms and late secretion of Th1/Th2/Th17 cytokines. Importantly, in both mouse strains, the early depletion of CD25^+^ cells resulted in less severe tissue pathology and abolished the enhanced mortality observed in susceptible mice. In conclusion, this study is the first to demonstrate that anti-CD25 treatment is beneficial to the progressive and regressive forms of paracoccidioidomycosis, potentially due to the anti-CD25-mediated reduction of Treg cells, as these cells have suppressive effects on the early T cell response in resistant mice and the clearance mechanisms of fungal cells in susceptible mice.

## Introduction

Paracoccidioidomycosis (PCM), the most important systemic mycosis in Latin America, is caused by the dimorphic fungus *Paracoccidioides brasiliensis* [1]. The inhalation of conidia usually leads to an asymptomatic infection but a few infected individuals evolve to overt disease.The infection and the benign forms of the disease are associated prevalent Th1 immunity whereas the severe forms with suppressed DTH responses and prevalent Th2/Th3 immunity [2], [3]. In a genetic model of infection, A/J mice was shown to be resistant while B10.A mice susceptible to the i.p and i.t. routes of infection [4], [5]. In the pulmonary model, A/J mice are initially tolerant to fungal growth but lately develop efficient cellular immunity with prevalent IFN-γ secretion, macrophage activation and regressive disease [6], [7]. In contrast, B10.A mice are able to control the initial infection but soon develop T cell anergy and disseminated disease [5], [8], [9]. Both, CD4^+^ and CD8^+^T cells were shown to be immunoprotective against *P. brasiliensis* infection. Resistant mice develop mixedTh1/Th2 responses concomitant with protective CD8^+^ T cells that synthesize large amounts of IFN-γ [7], [8], [10]. The relative protection of susceptible mice is mediated by CD8^+^ T cells which, however, are not able to compensate the CD4^+^ T cell anergy induced by excessive nitric oxide (NO) production [7], [11] and indoleamine-2,3 dioxygenase (IDO) activity [Araujo et al., manuscript in preparation].

CD4^+^CD25^+^ regulatory T cells (Treg) have been shown to control the afferent and efferent arms of immune responses, and play an essential role in the control of autoimmune diseases, transplantation and infectious processes [12]–[15]. Natural Treg cells develop in the thymus, have a CD4^+^CD25^+^ phenotype, and are seeded to peripheral lymphoid organs where they control autoimmunity and excessive inflammatory responses against endogenous and exogenous aggressions [16], [17]. At the periphery, naïve CD4^+^ T cells can also acquire a suppressive phenotype and ability to control excessive immunity [17], [18]. In addition to CD25 (the alpha chain of IL-2R), Treg cells express other activation markers such as CTLA-4 (CD152, cytotoxic T lymphocyte-associated antigen 4), GITR (glucocorticoid-induced tumor necrosis factor-receptor-related protein), OX40 (CD134), and L-selectin or CD62 ligand (CD62L) [19]–[21]. Several transcription factors were shown to control Treg cells development and activity, but Foxp3 has been described as the crucial factor for the suppressive function of these cells [22]–[25]. The suppressive activity of Tregs depends on cell contact and/or the activity of several inhibitory molecules such as IL-10, TGF-β, IL-35, CTLA-4, IDO, and granzyme/perforin [18], [22], [26]. Although Tregs are likely to use multiple mechanisms to suppress immune responses, CTLA-4 may have a dominant role [27]–[29]. There is increasing evidence that Treg cells and, in particular, natural CD4^+^CD25^+^ Treg cells play a key role in the control of infectious processes. The presence of Treg cells has been associated with many chronic infectious diseases where they facilitate the maintenance of a residual number of microorganisms and immunological memory [14], [17], [30]. Treg cells were shown to increase fungal loads in mice infected with *Candida albicans, Pneumocystis carinii* and *Aspergillus fumigatus*. In these experimental models, while maintaining infection, Treg cells limited the associated immunopathology [31]–[34]. There are few reports in the literature on the participation of Treg cells in the control of paracoccidioidomycosis, but increased numbers of these cells have been associated with disease severity. Thus, a higher number of Foxp3^+^ Treg cells was demonstrated in the lesions and peripheral blood of patients with active disease when compared with treated patients or healthy controls. These cells produced high levels of TGF-β and IL-10 and expressed increased levels of characteristic surface markers of Tregs (GITR, CD38, CD95L, CTLA-4, TLR-2 and LAP) [35], [36]. Furthermore, in CCR5-deficient C57BL/6 mice, which have intermediate susceptibility to *P. brasiliensis* infection, the survival of yeast cells and the severe immunosuppression of hosts were shown to be mediated by Treg cells [37]. In the pulmonary model of murine PCM, our group recently showed that the development of Treg cells was associated with CD28, TLR2 and TLR4 expression. [38]–[40]. In addition, the adaptor protein MyD88 was also shown to be involved in the control of Treg cells differentiation [41].

In this study we explored the presence, phenotype and function of CD4^+^CD25^+^Foxp3^+^ Treg cells in resistant A/J and susceptible B10.A mice to *P. brasiliensis* infection. Subsequently, the severity of the disease was studied at an early and late periods of infection using anti-CD25-treated and untreated mice. Interestingly, uninfected and infected resistant mice presented higher numbers and more potent Treg cells than susceptible mice. The early depletion of CD25^+^ cells by monoclonal antibodies led to a less severe infection in both mouse strains, but only in resistant mice the early migration of inflammatory cells to the site of infection was restored. Antibody-mediated depletion of CD25^+^ T cells of susceptible did not alter the migration of inflammatory T cells, but recued these animals from progressive disease and precocious mortality. Importantly, anti-CD25 treatment did not induce sterile immunity, but significantly reduced organ pathology. In conclusion, ours results showed for the first time regulatory T cells exert detrimental effects to resistant and susceptible mice to *P. brasiliensis* infection, and their modulation by anti-CD25 treatment can bring beneficial effects to both, the progressive and regressive forms of this chronic fungal disease.

## Materials and Methods

### Ethics Statement

Animal experiments were performed in strict accordance with the Brazilian Federal Law 11,794 establishing procedures for the scientific use of animals, and the State Law establishing the Animal Protection Code of the State of São Paulo. All efforts were made to minimize suffering, and all animal procedures were approved by the Ethics Committee on Animal Experiments of the Institute of Biomedical Sciences of University of São Paulo (Proc.76/04/CEEA).

### Mice

A/J (resistant), B10.A (susceptible), and Foxp3^tm1Kuch^ C57BL/6 (intermediate susceptibility) mouse strains were bred at the University of São Paulo animal facilities under specific-pathogen-free (SPF) conditions in closed-top cages. The Foxp3^GFP^ reporter allele C57BL/6 mouse strain was kindly donated by Dr.Vijay K. Kuchroo, from Harvard University. Clean food and water were given ad libitum. Mice were 8 to 11 weeks of age at the time of infection, and procedures involving animals and their care were approved by the Ethics Committee on Animal Experiments from Instituto de Ciências Biomédicas, Universidade de São Paulo.

### Fungus and Mice Infection

*P. brasiliensis* 18 isolate (Pb18), which is highly virulent, was used throughout the study. To ensure the maintenance of its virulence, the isolate was used after three serial animal passages [42]. Pb18 yeast cells were then maintained by weekly subcultivation in semisolid Fava Netto culture medium [43] at 35°C and used on the seventh day of culture. The fungal cells were washed in phosphate-buffered saline (PBS; pH 7.2) and counted in a hemocytometer and the suspension was adjusted to 20×10^6^ fungal cells/mL. The viability of fungal suspension, determined by Janus Green B vital dye (Merk, Darmstadt, Germany), was always higher than 85%. Mice were anesthetized and submitted to i.t. *P. brasiliensis* infection as previously described [5]. Briefly, after intraperitoneal anesthesia the animals were infected with 1×10^6^ Pb18 yeast cells, contained in 50 µL of PBS, by surgical i.t. inoculation, which allowed dispensing of the fungal cells directly into the lungs. The skins of the animals were then sutured, and the mice were allowed to recover under a heat lamp. Mice were studied 2 and 10 weeks after infection. Two to three experiments were performed separately.

### *In vivo* Depletion of CD25^+^ T cells

B10.A, A/J mice and Foxp3^GFP^ C57BL/6 mice were given i.p. injections of 0.5 mg of an anti-CD25 (clone PC61) or a control rat IgG obtained from BioXcell (USA). Antibodies were administered 3 days before and 3 days after infection with *P. brasiliensis* yeasts. Foxp3^GFP^ C57BL/6 mice were studied at week 2 post-infection. A/J and B10.A mice were sacrificed 2 and 10 weeks after infection, and organs analyzed for CFU counts, histopathology, inflammatory reactions and levels of cytokines. PC61 administration promoted long-lasting depletion/neutralization of CD25 expression (longer than 2weeks) in naïve mice, as previously demonstrated [44], [45].

### Assay for Organ Colony Forming Units (CFU)

The number of viable microorganisms in infected organs (lung, liver and spleen) from experimental and control mice were determined by counting the number of CFU. Animals (n = 6–8) from each group were sacrificed, and the enumeration of viable organisms was done as previously described [46]. Briefly, aliquots (100 µL) of the cellular suspensions and serial dilutions were plated on brain heart infusion agar (Difco, Detroit, MI) supplemented with 4% (vol/vol) horse serum (Instituto Butantan, São Paulo, Brazil) and 5% *P. brasiliensis* 192 culture filtrate, the latter constituting a source of growth-promoting factor. The plates were incubated at 35°C, and colonies were counted daily until no increase in counts was observed. The number (log10) of viable *P. brasiliensis* colonies per gram of tissue were expressed as means ± standard errors (SE).

### Mortality Rates

Mortality studies were performed with anti-CD25-treated and control A/J and B10.A mice inoculated i.t. with 1×10^6^ yeast cells or PBS (n = 9–11). Deaths were registered daily for a 200-day period and experiments were repeated twice.

### Lung and Liver Leukocytes Isolation

Lungs from each mouse were excised, washed in PBS, minced, and digested enzymatically for 1 hour in 15 mL/lung of digestion buffer [RPMI, 5% fetal calf serum, 1 mg/mL collagenase (Sigma Aldrich Inc.®), and 30 µg/mL DNase]. Livers from individual mice were obtained, submitted to organ perfusion using 10.0 ml of warm PBS via portal vein and organ fragments were pressed through 70 µm cell strainer (Becton Dickson). After erythrocyte lysis using NH4Cl buffer, cells were washed, resuspended in complete media, and centrifuged for 30 minutes at 1,200×g in presence of 20% Percoll (Sigma) to separate leukocytes from cell debris and epithelial cells. Total leukocyte numbers were assessed in the presence of trypan blue using a hemocytometer; viability was always higher than 85%. The absolute number of a leukocyte subset was equal to the percentage of that cell subset multiplied by the total number of leukocytes recovered from the digested organ/100.

### Purification of Cells

Magnetic sorting of various splenic and lung cell types were performed with the AutoMACS (MiltenyiBiotec, Germany), according to the manufacturer’s instructions. Splenic effector CD4^+^CD25^−^ T cells from naive B10.A and A/J mice were sorted after labeling cells with the CD4^+^ selection MACS kit. Suppressor CD4^+^CD25^+^ T cells were obtained from lung cell suspensions previously prepared as above described. Using the CD4^+^CD25^+^ isolation kit, non-CD4^+^ cells were depleted first using antibodies targeting CD8^+^ T cells, B cells, macrophages, NK cells and erythrocytes. CD25^+^ cells were then positively selected using PE-labeled anti-CD25 and anti-PE microbeads.

### Coculture Proliferation-suppression Assay

Responder CD4**^+^** T cells from naïve B10.A and A/J mice were stained with CFSE (Invitrogen). To perform the cell staining, 5×10^6^ cells were incubated with 10 µM CFSE in RPMI containing 5% FBS for 15 min at 37°C in the dark. Cells were then washed twice and resuspended in RPMI containing 10% FBS for seeding. CD4^+^CD25^−^ T cells (5×10^4^/well) were seeded in U-bottom, 96 well plate in RPMI with 10% FBS, containing 2 mM glutamine, 100 IU/penicillin, 100 mg/ml streptomycin, 10 mM Hepes, 1 mM sodium pyruvate, and 50 µM 2-ME, with or without 2×10^5^ cell/well T-cell depleted, irradiated spleen cells, plus 0.5 µg/ml soluble anti-CD3 antibody. CD4^+^CD25^+^ T cells from infected lungs were added to the well at a ratio (CD4^+^CD25^−^ T cell: CD4^+^CD25^+^ T cell) of 1∶1, 1∶0.33 and 1∶0.11. Cells were cultivated for 5 days and the proliferative response of CFSE-labeled cells was measured by flow cytometry (FacsCanto). The proliferation index (PI) was calculated dividing the geometric mean of fluorescence from non-stimulated CD4^+^CD25^−^ T cells by the geometric mean of fluorescence from stimulated CD4^+^CD25^−^ T cells in the presence or absence of CD4^+^CD25^+^ T cells.

### Flow Cytometry Analysis

For surface staining alone, lung or liver leukocytes were washed and resuspended at a concentration of 1×10^6^ cells/mL in staining buffer (PBS 1x, 2% FBS and 0,5% NaN3). Fc receptors were blocked by the addition of unlabeled anti-CD16/32 (Fc block; BD Pharmingen, San Diego, CA). The leukocytes were then stained for 20 min at 4°C with the optimal dilution of each antibody labeled with the adequate fluorochrome (BD, Pharmingen). The following antibodies were used: anti-CD11b, CD11c, F4/80, GR1, Iak, CD3, CD4, CD8, CD44, CD62L, CD25, CTLA-4, GITR, LAP, CD19, and B220. Cells were washed twice with staining buffer, resuspended in 100 µl, and an equal volume of 2% formalin was added to fix the cells. The stained cells were analyzed immediately on a FACSCanto flow cytometer (BD Biosciences, CA) using the FACSDiva software (BD Biosciences) gating on macrophages or lymphocytes as judged from forward and side light scatter. Twenty thousand cells were counted and the data expressed as the percentage or the absolute number of positive cells which was calculated trough the percentage obtained by FACS and the number of cells determined in Neubauer chambers. The intracellular detection of Foxp3, the X-linked forkhead/winged helix transcription factor, in leukocytes obtained from infected lung or liver was performed in fixed and permeabilized cells using Cytofix/Cytoperm (BD Biosciences). Initially, the cells were labeled with antibodies for cell surface molecules such as FITC-conjugated anti-CD4 and PE-Cy7 conjugated anti-CD25. Next, the cells were fixed, permeabilized, and stained with PE-conjugated anti-Foxp3, for 90 min at 4°C. In selected experiments, Foxp3+ Treg cells were also labeled for membrane LAP, CTLA4 and GITR expression. For intracellular cytokines, after Foxp3 labeling (PE or APC), pacific blue anti IL-10 and PE anti-TGF-β were added to fixed and permeabilized cells. Cells were then washed twice with staining buffer, resuspended in 100 µl, and an equal volume of 2% formalin was added to fix the cells. A minimum of 100,000 events were acquired on FACSCanto flow cytometer using the FACSDiva software, as described above. Surface staining of CD25 and intracellular Foxp3 expression were back-gated on the CD4 T cell population.

### Measurement of Cytokines

Mice were infected i.t. with *P. brasiliensis* (n = 6–8), and their right lung was aseptically removed and individually disrupted in 5.0 mL of PBS. Supernatants were separated from cell debris by centrifugation at 2,000×g for 15 min, passed through 0.22 µm pore-size filters (Millipore, Bedford, MA), and stored at −70°C. The levels of IL-2, IL-12, IFN-γ, TNF-α, IL-4, IL-10, GM-CSF, IL-6, IL-23, IL-17 and TGF-β were measured by capture enzyme-linked immunosorbent assay (ELISA) with antibodies pairs purchased from Pharmingen. The ELISA procedure was performed according to the manufacture’s protocol. The concentrations of cytokines were determined with reference to a standard curve for several twofold dilutions of murine recombinant cytokines.

### Histopathologic and Morphometrical Analyzes

Groups of anti-CD25 treated and untreated B10.A and A/J mice were killed at the second and tenth weeks post-infection. Lungs and livers were collected, fixed in 10% formalin and embedded in paraffin. Five-micrometer sections were stained by the hematoxilin-eosin (H&E) for an analysis of the lesions, silver stained for fungal evaluation. Pathological changes were analyzed based on the size, morphology and cell composition of granulomatous lesions, presence of fungi and intensity of the inflammatory infiltrates. Morphometrical analysis was performed using a Nikon DXM 1200c digital camera (magnification of 10x), and Nikon NIS Elements AR 2.30 software. The area of lesions was measured (in µm2) in 10 microscopic fields per slide (n = 4–6). Results were expressed as the mean ± standard error of the mean (SEM) of total area of lesions for each animal.

### Quantitative Analysis of 2,3 Indoleamine Dioxygenase (IDO) mRNA Expression

RNA was extracted from normal and infected lungs using Trizol reagent (Invitrogen,Calrsbad, CA), and cDNA was synthesized from 1 mg RNA using High Capacity RNA-to-cDNA kit (Applied Biosystems) according to manufacturer’s instructions. IDO mRNA expression was quantified relative to glyceraldehyde-3-phosphate dehydrogenase (GAPDH) using assay-on-demand primers and probes, Taqman Universal Master Mix, and ABI Prism 7000 apparatus (Applied Biosystems).

### Determination of IDO Enzymatic Activity

To monitor IDO enzymatic activity, kynurenine was detected using a modified spectrophotometric assay. The amount of 50 µl of 30% trichloroacetic acid was added to 100 ml of lung homogenates, vortexed, and centrifuged at 800 *g* for 5 min. A volume of 75 µl of the supernatant was then added to an equal volume of Ehrlich reagent (100 mg P-dimethylbenzaldehyde, 5 ml glacial acetic acid) in a 96 wells microtiter plate. Optical density was measured at 492 nm, using a Multiskan MS (Labsystems, Helsinki, Finland) microplate reader. A standard curve of defined kynurenine concentrations (0–100 mM) was used to determine unknown kynurenine concentrations.

### Statistical Analysis

Data are expressed as the mean ± SEM. Differences between groups were analyzed by Student's *t* test or analysis of variance (ANOVA) followed by the Bonferroni test. Differences between survival times were determined with the LogRank test. Data were analyzed using GraphPad Prism 5.01 software for Windows (GraphPad, San Diego, CA). P values ≤0.05 were considered significant.

## Results

### Early in the Infection, Resistant Mice Develop a More Severe Infection and Present a Higher Number of Treg Cells in the Lungs

**Figure 1:**
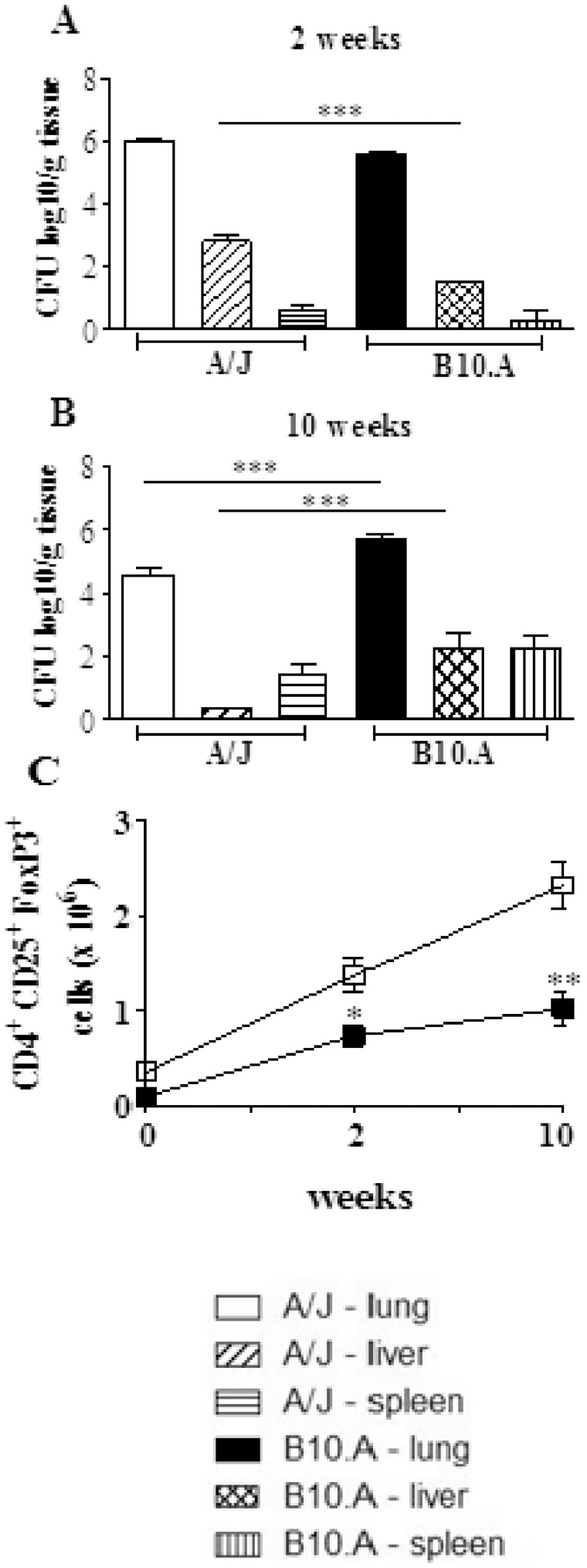
In the course of *P.brasiliensis* infection resistant (A/J) mice develop higher numbers of Treg cells than susceptible (B10.A) mice. Groups (n = 6) of A/J and B10.A mice were infected i.t. with 1×10^6^ yeasts cells of *P.brasiliensis*. Organ CFU counts were measured at weeks 2 (A) and 10 (B) after infection. At these time points, lungs were removed, leukocytes obtained and the number of CD4^+^CD25^+^Foxp3^+^ cells analyzed by flow cytometry. The data represent the mean ± SEM of the results from 6 mice per group and are representative of two independent experiments. * (*P*<0.05), ** (*P*<0.01), and *** (*P*<0.001) compared with A/J mice.

First we characterized the severity of *P.brasiliensis* infection in resistant (A/J and susceptible mice (B10.A) infected i.t. with 1×10^6^ viable yeast cells. This control was necessary because there is no adequate virulence marker for *P. brasiliensis* whose infectivity is maintained by in vivo passages, resulting in virulence fluctuation. The data here obtained are consistent with those previously described [5]. At week 2, the pulmonary infection of A/J mice is slightly higher than in B10.A mice, and allows an early dissemination and enhanced fungal growth in the liver (Fig. 1A). At week 10, the infection is regressive in A/J mice and progressive in B10.Amice. At this late period, while A/J mice control the fungal growth in the liver, B10.A mice show increased numbers of viable yeasts in the liver and spleen (Fig. 1B). These data reproduce our earlier findings in the pulmonary model of infection, and demonstrate that, early in the infection, A/J mice are more permissive to fungal growth and dissemination than the susceptible strain. However, at the late phase A/J mice develop an adequate control of the infection whereas a progressive disease is established in B10.A mice.

As Treg cells were shown to be involved in the control of several infectious processes [14], [17], [30]–[34], we asked whether these cells could be associated with the resistant and susceptible phenotype of A/J and B10.A mice. We have then assessed the presence of regulatory T cells in the lungs of A/J and B10.A mice infected with 1 million fungal cells by the pulmonary route. In the course of infection, both mouse strains increased the numbers of pulmonary CD4^+^CD25^+^Foxp3^+^ Treg cells. However, compared with B10.A mice, and at both post-infection periods, higher numbers of CD4^+^CD25^+^Foxp3^+^ Treg cells were found in A/J mice (Fig. 1C). Interestingly, the total number of mononuclear leukocytes present in the lungs of uninfected B10.A mice was 1.8 fold higher than those of A/J mice (44.8±5.2×10^6^ cells X 24.5±2.7×10^6^ cells). However, even uninfected A/J mice (day zero) showed an augmented number (0.36±0.04×10^6^ X 0.10±0.02×10^6^ cells) and frequency (1.46±0.16 X 0.2±0.02) of pulmonary Treg cells than susceptible B10.A mice.

### Number and Phenotype of Regulatory T cells in the Course of *P. brasiliensis* Infection of Resistant and Susceptible Mice

**Figure 2:**
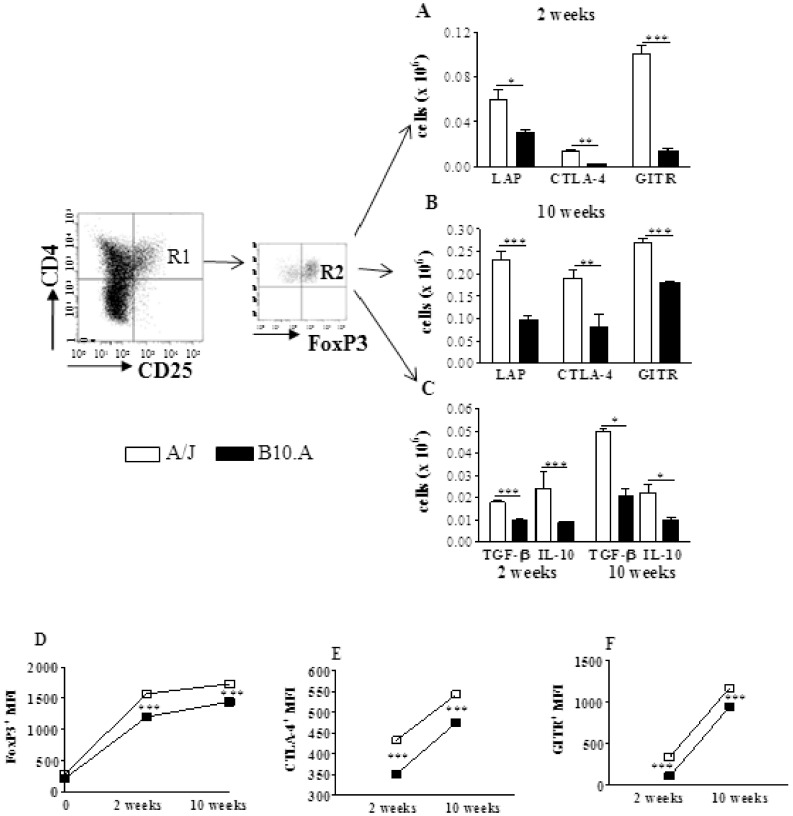
Characterization of Treg cells: resistant A/J mice present increased numbers of Treg cells expressing higher levels of Foxp3, CTLA4, and GITR. Characterization of pulmonary Treg cells by flow cytometry in the lung infiltrating leucocytes (LIL) from A/J and B10.A mice inoculated i.t. with 1×10^6^ *P. brasiliensis* yeast cells. At weeks 2 and 10 after infection lung cell suspensions were obtained and stained as described in Materials and Methods. The stained cells were analyzed immediately on a FACS CANTO equipment gating on lymphocytes as judged from forward and side light scatters. Representative FACS plots demonstrating the gates used to characterize Treg cells (R1, percentage of CD4^+^CD25^+^ cells in lymphocytes gate, and R2, percentage of Foxp3^+^ cells within the CD4^+^CD25^+^ population). Number of CD4^+^CD25^+^Foxp3^+^ cells expressing LAP, CTLA4 and GITR at weeks 2 (A) and 10 (B) of infection. C- Absolute numbers of CD4^+^CD25^+^Foxp3^+^ cells expressing intracellular TGF-β and IL-10. The relative expression of Foxp3 by CD4^+^CD25^+^ cells (D) and the relative expression of CTLA4 (E) and GITR (F) by CD4^+^CD25^+^Foxp3^+^ cells at the indicated time points after *P.brasiliensis* infection. The data represent the mean ± SEM of the results from 6 mice per group and are representative of two independent experiments. * (*P*<0.05), ** (*P*<0.01), and *** (*P*<0.001) compared with A/J mice.

To better characterize CD4^+^CD25^+^Foxp3^+^ Treg cells, the expression of membrane TGF-β (LAP), CTLA4, GITR and intracellular TGF-β and IL-10 were evaluated. As shown in (Fig. 1A,B), A/J mice showed increased numbers of Treg cells expressing all studied activation markers, besides increased presence of intracellular IL-10 and TGF-β (Fig. 1 C). As the expression of Foxp3 and CTLA4 have been implicated in the suppressive potency of Treg cells (16,29) we analyzed the mean fluorescence intensity of these molecules expressed by Treg cells. As shown in Fig. 2D–F, the intensity of all these three molecules increased in the course of infection, and was always higher in A/J than in B10.A Treg cells.

### Regulatory T cells from Resistant Mice have a More Potent Suppressive Activity

**Figure 3:**
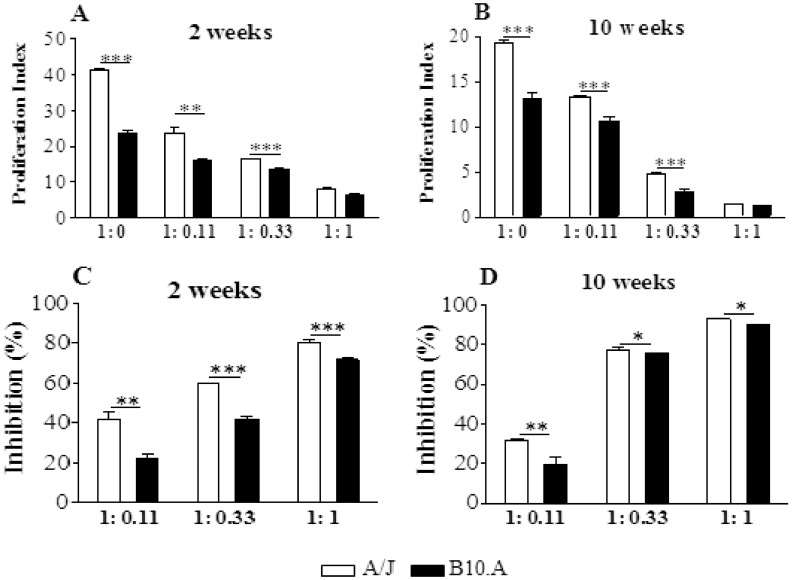
Treg cells from resistant mice have a higher suppressive potency than those of susceptible mice. CFSE-labeled responder CD4^+^CD25^−^ T cells from naïve mice were stimulated by irradiated naïve APCs plus anti-CD3 antibodies and cultured in the presence or absence of several ratios of CD25^+^ T cells obtained from lungs of resistant and susceptible mice at weeks 2 (A) and 10 (B) after infection with 1×10^6^ *P.brasiliensis* yeasts. Cells were cultured for 5 days and the proliferative response of CFSE-labeled cells was measured by flow cytometry. The proliferation index (PI) was calculated as describe in materials and methods and the percentage of inhibition considered as 100% the PI of APC-stimulated CD4^+^CD25^−^ responder cells in the absence of CD4^+^CD25^+^ cells. The data represent the mean ± SEM of the results from 6 mice per group and are representative of two independent experiments. * (*P*<0.05), ** (*P*<0.01), and *** (*P*<0.001) compared with A/J mice.

Following verifying that resistant mice had higher numbers of Treg cells expressing higher levels of Foxp3 and CTLA4 in their lungs than B10.A mice, we asked whether these cells had equivalent suppressive activity. Then, naïve CD4^+^CD25^−^splenic T cells from uninfected mice were stimulated by irradiated APCs plus anti-CD3 antibodies in the presence or absence of CD4^+^CD25^+^ T cells isolated from the lungsof B10.A and A/J mice at weeks 2 and 10 after infection. CD4^+^CD25^−^ cells were previously labeled with CFSE, stimulated for 5 days, and the proliferative response analyzed by flow cytometry. The results are expressed as proliferation index (PI) and percentage of inhibition. At both infection periods, naïve spleen cells from A/J mice showed an increased proliferative response, and the addition of several proportions of Treg cells led to decreased proliferation, which was maximal at the T effector:Treg ratio of 1∶1 (Fig. 3A, B). When the percentage of inhibition was calculated, we could see that Treg cells from A/J mice exerted a superior suppressive activity on lymphocyte proliferation than B10.A Tregs. Importantly, this difference between strains was most marked at the second than at the ten^th^ week after infection. At week 10, however, Treg cells appear to be more suppressive than those obtained at week 2, and this fact could be seen with the higher proportions of Treg cells employed (Fig. 3 C,D). As a whole, these findings demonstrated that in the course of infection resistant and susceptible mice develop increased numbers of Treg cells with increased suppressive potency. However, Tregs from resistant mice have a more potent suppressive activity on the proliferation of naïve T cells than those of B10.A mice.

**In Resistant and Susceptible Mice, Depletion of CD25^+^ T Cells Promotes an Enhanced Control of Fungal Growth.**

**Figure 4:**
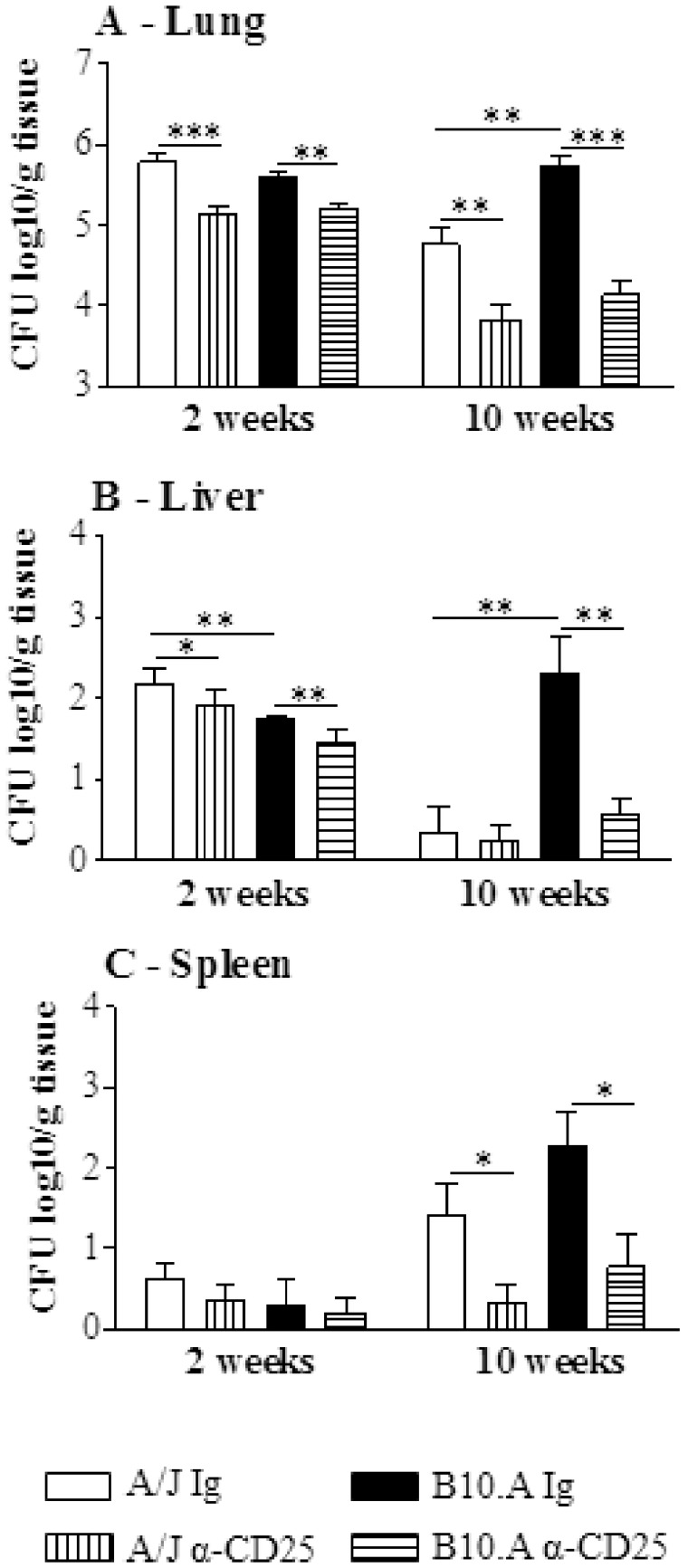
Administration of anti-CD25 antibody decreases the early and late organ fungal burdens of resistant and susceptible mice. Anti-CD25 mAb (PC61) or control IgG (500 µg) were administered at days −3 and +3 of *P. brasiliensis* infection. CFU counts were determined in the lungs (A), liver (B) and spleen (C) of resistant and susceptible mice at weeks 2 and 10 after infection. The points represent means ± SEM of log_10_ CFU obtained from groups of six mice. The results are representative of 3 experiments * (*P*<0.05), ** (*P*<0.01), and *** (*P*<0.001) compared with IgG inoculated controls.

We next asked whether anti-CD25 treatment of A/J and B10.A mice would change the severity of fungal infection. This treatment was chosen because it induces a consistent and persistent (2 weeks) depletion of Treg cells [44]. Then, groups (n = 6–7) of susceptible and resistant mice were treated i.p. with 500 µg of anti-CD25 monoclonal antibody (PC61) at days −3 and +3 of *P.brasiliensis* infection. Control mice were injected with an equivalent amount of normal rat IgG. In both mouse strains, at both post-infection periods, a decreased fungal burden was observed. Of note was the effect anti-CD25 administration at the late period of infection of susceptible mice. Besides the lowered CFU counts in the lungs, the livers and spleens of B10.A mice showed an impressive reduction in the number of viable yeast cells (Fig. 4A–C). I.

**Figure 5:**
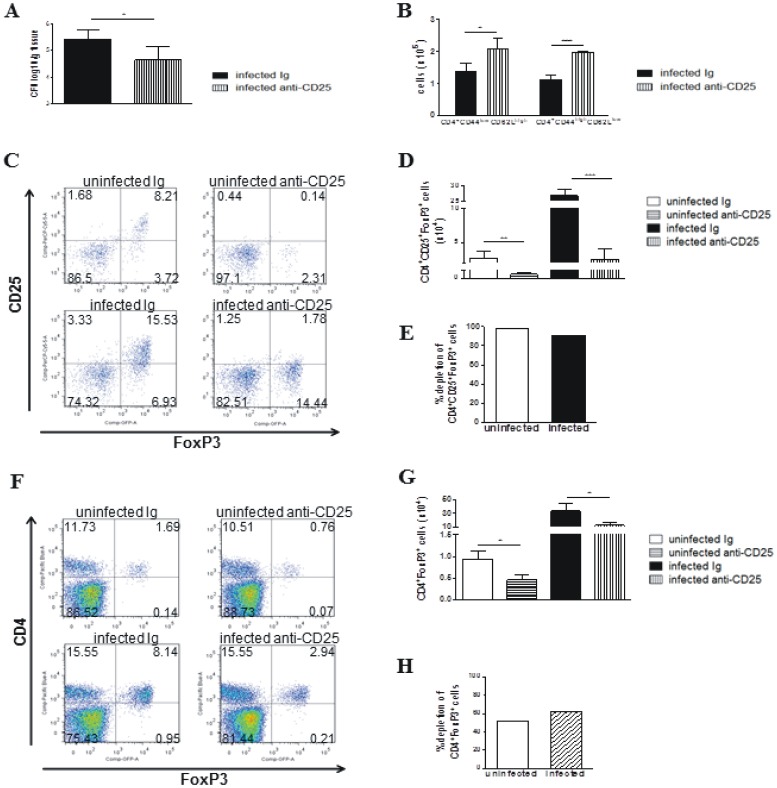
Anti-CD25 treatment depletes Foxp3^GFP^ Treg cells and induces decreased fungal loads in Foxp3^GFP^ C57BL/6 mice. Anti-CD25 antibodies (PC61) were administered as described in materials and methods to uninfected and *P.brasiliensis* infected (1×10^6^, i.t. route) Foxp3^GFP^ C57BL/6 mice. Normal Ig was injected in uninfected and infected control groups. Fungal loads (A), number of naïve (CD4^+^CD44^low^CD62L^high^) and activated (CD4^+^CD44^high^CD62L^low^) CD4^+^ T cells (B), number of CD4^+^CD25^+^Foxp3^GFP^ Treg cells (D), percentage depletion of CD4^+^CD25^+^Foxp3^GFP^ Treg cells (E), number of CD4^+^Foxp3^GFP^ Treg cells (G) and percentage depletion of CD4^+^ Foxp3^GFP^ Treg cells (E) were assessed in lung inflammatory infiltrates at week 2 after infection. Data are from a single experiment with 5–6 mice per group. Cell phenotypes were assessed by flow cytometry. Data represent mean ± SEM. * (*P*<0.05), ** (*P*<0.01), and *** (*P*<0.001) between groups.

In an attempt to better characterize the effect of anti-CD25 (PC61) treatment on normal and *P. brasiliensis*-infected mice, we performed some experiments using Foxp3^GFP^ C57BL/6 mice. The groups of uninfected and infected mice (1×10^6^ *P. brasiliensis* injected through the i.t. route) were treated or not treated with anti-CD25 (PC61) mAb using the same protocol that was used to treat the A/J and B10.A mice. As shown in Figure 5, decreased fungal burdens (Fig. 5A) and Foxp3^+^ Treg cells (CD4^+^CD25^+^Foxp3^+^), which were associated with increased numbers of effector CD4 T (CD44^high^CD62^low^) cells, were detected at week 2 post-infection (Fig. 5B–E). In addition, the FoxP3^+^ cells were characterized by the expression of GFP and not by the use of anti-CD25 antibodies. Although less extreme than that observed with the anti-CD25 antibodies, a decreased number of FoxP3^+^ cells was observed (Fig. 5F–H). Therefore, the anti-CD25 antibodies induced a consistent decrease in Foxp3^GFP^ Treg cells that was associated with an increase (not a decrease) in effector T cells. These data brought consistent evidence that treatment with PC61 antibodies effectively depletes Treg cells and increases effector T cells in *P. brasiliensis* infected mice.

### Depletion of CD25^+^ Cells Affects the Number but not the Frequency of Pulmonary Leukocytes

**Figure 6:**
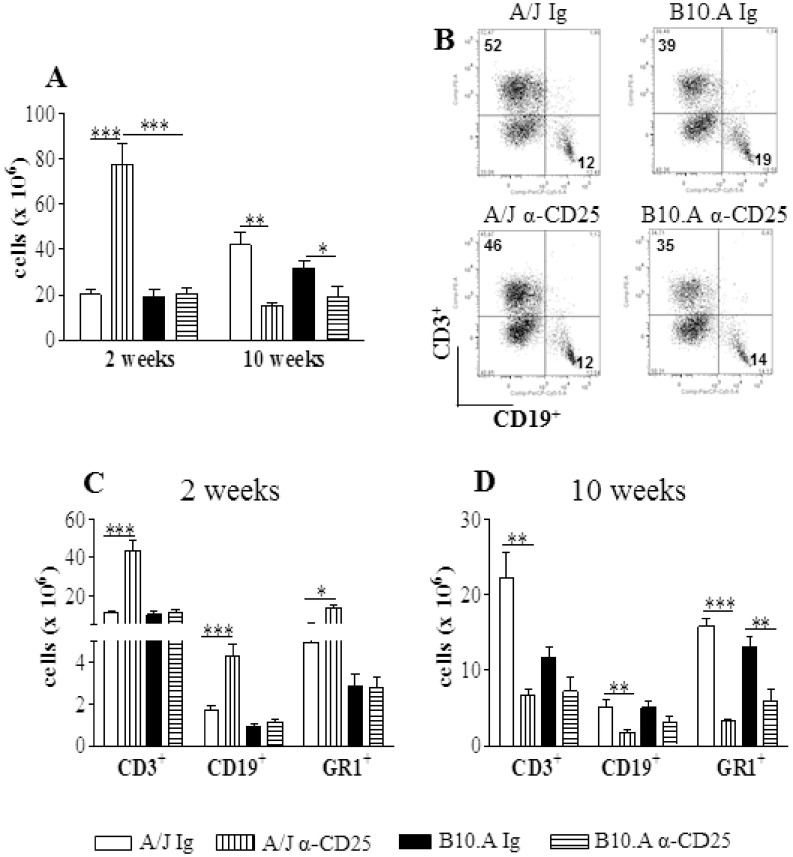
Anti-CD25 treatment increases the influx of inflammatory cells to the lungs of resistant but not susceptible mice to *P. brasiliensis* infection. Anti-CD25-treated and untreated A/J and B10.A mice were inoculated i.t. with 1×10^6^ *P. brasiliensis* yeast cells. At weeks 2 and 10 of infection lungs of both mouse strains (n = 6) were excised, minced, and digested enzymatically. Lung cells suspensions were obtained, counted, stained for CD3 (T cells), CD19 (B cells) and GR1 (myeloid cells, including neutrophils and monocytes) by flow cytometry. Anti-CD25 treatment significantly alters the number (A) but not the frequency of inflammatory cells in the lungs (B) of infected mice. At week 2, anti-CD25-treated A/J mice showed increased influx of T cells, B cells and myeloid cells, whereas at week 10 these populations appeared in decreased numbers (C, D). In B10.A mice only GR1^+^ cells appeared in decreased numbers at week 10 of infection (C, D). The data represent the mean ± SEM of the results from 6–8 mice per group and are representative of two independent experiments. * (*P*<0.05), ** (*P*<0.01), and *** (*P*<0.001) compared with IgG-treated mice.

In the lungs of A/J mice, anti-CD25 treatment increased the number of inflammatory cells at week 2, but led to diminished numbers at week 10 of infection. In contrast, in B10.A mice a reduced inflammation was seen only at week 10 (Fig. 6A).When the frequency of T cells, B cells and GR1^+^ cells (myeloid cells, including cells of the granulocytic lineage such as neutrophils and the monocytic lineage) was characterized no important differences between treated and untreated groups were observed (Fig. 6B); however, A/J mice showed all leucocyte subsets in increased numbers at week 2 and reduced at week 10 of infection. In B10.A mice, only GR1^+^ cells were seen in decreased numbers at the late phase of infection (Fig. 6C, D).

### Anti-CD25 Treatment Increases the Early Influx of T cells to the Lungs of Resistant but not of Susceptible Mice

**Figure 7:**
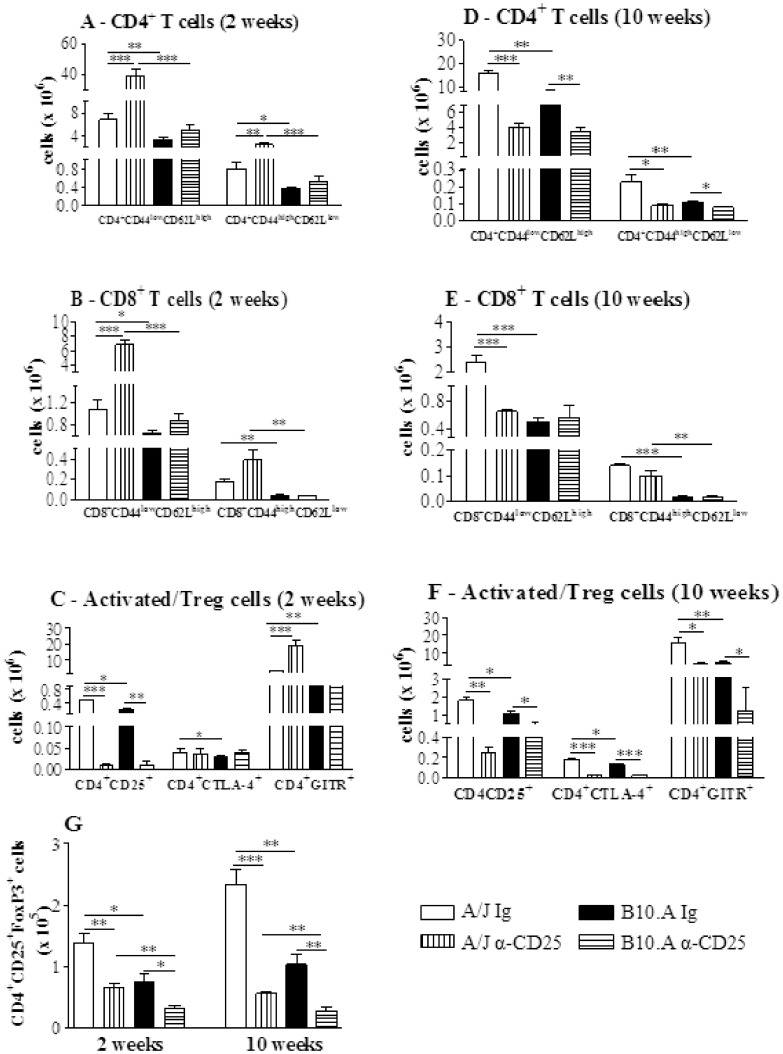
Anti-CD25 causes intense alterations in the migration of inflammatory CD4^+^ and CD8^+^ T cells to the lungs of A/J mice, but does not affect B10.A mice. Characterization of CD4^+^, CD8^+^ T cells and activation profile of cells by flow cytometry in the lung infiltrating leucocytes (LIL) from anti-CD25-treated and untreated A/J and B10.A mice inoculated i.t. with 1×10^6^ *P. brasiliensis* yeast cells. At weeks 2 and 10 after infection lung cell suspensions were obtained and stained as described in Materials and Methods. The acquisition and analysis gates were restricted to lymphocytes. CD4^+^ T cells (A), CD8^+^ T cells (B) and activated/Treg (C) cells at week 2 of infection. CD4^+^ T cells (D), CD8^+^ T cells (E) and activated/Treg (F) cells at week 10 of infection. To characterize the number of Treg cells in LIL, surface staining of CD25^+^ and intracellular Foxp3 expression were back-gated on the CD4^+^ T cell population (G). The data represent the mean ± SEM of the results from 6–8 mice per group and are representative of two independent experiments. * (*P*<0.05), ** (*P*<0.01), and *** (*P*<0.001) compared with IgG treated controls or the susceptible strain.

The early depletion of CD25^+^ cells from A/J mice profoundly affected the influx of inflammatory T cells to their lungs. Increased numbers of CD4^+^ and CD8^+^ naïve (CD4^+^CD44^low^CD62L^high^ and CD8^+^CD44^low^CD62L^high^, respectively) as well as CD4^+^ and CD8^+^ effector (CD4^+^CD44^high^CD62L^low^ and CD8^+^CD44^high^CD62L^low^) T cells were detected at week 2 after infection. In contrast, no alterations in the early influx of inflammatory T cells to the lungs of B10.A mice were noticed (Fig. 7A–B). When other activation markers of T cells were studied, only the CD4^+^CD25^+^ subpopulation appeared in decreased number in anti-CD25-depleted groups, whereas CD4^+^GITR^+^ T cells were present in increased numbers only in A/J mice. No differences were detected in the CD4^+^CTLA4^+^ T cells (Fig. 7C). By week 10, almost all T cell phenotypes were present in decreased numbers in the lungs of resistant and susceptible mice (Fig. 7D–F). Thus, in A/J mice CD4 naïve and effector, CD8 naive, CD4^+^CD25^+^, CD4^+^CTLA4^+^, CD4^+^GITR^+^ cells were found in decreased numbers (Fig.7D–F). In B10.A mice, a decreased migration of CD4 naïve and effector T cells were observed in the lungs, but no differences in CD8^+^ T cells were found (Fig. 7D–F). In addition, CD4^+^ T cells expressing CD25, CTLA4 and GITR were also present in decreased numbers in the lungs of anti-CD25-treated B10.A mice (Fig. 7F).

To verify if anti-CD25 administration affected the presence of Treg cells in the lungs, the number of CD4^+^CD25^+^ T cells expressing Foxp3 was assessed. Indeed, despite the early interruption (day 3 post-infection) of anti-CD25 administration, a sustained depletion of Foxp3^+^ Treg cells was detected in the lungs of both mouse strains at both post-infection periods studied (Fig. 7G).

### The Early Depletion of CD25^+^ Cells Promotes an Increased Influx of Activated Macrophages and Dendritic Cells to the Lungs of Resistant Mice

**Figure 8:**
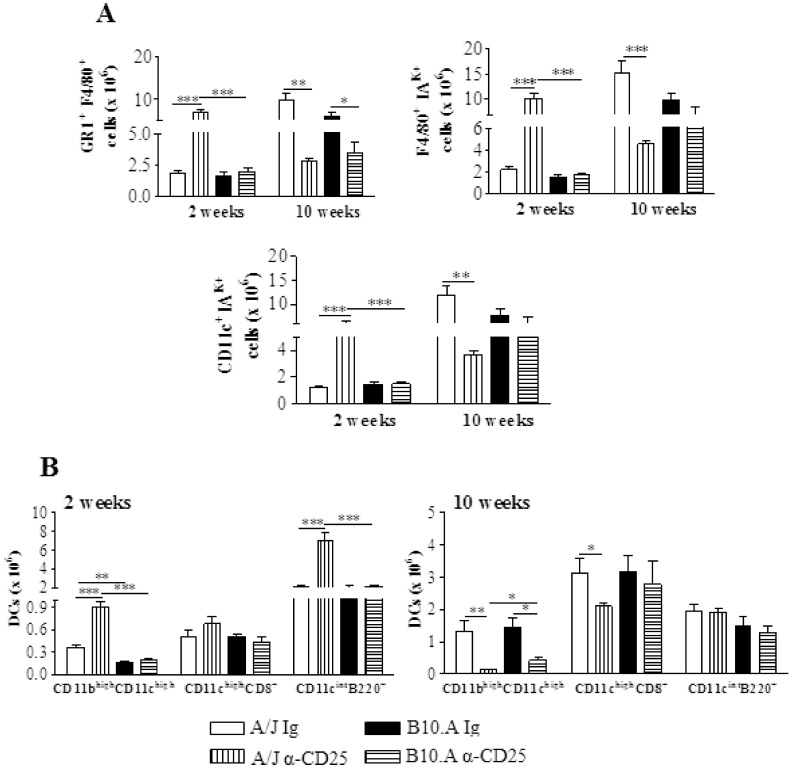
Anti-CD25 causes intense alterations in the migration of macrophages and dendritic cells to the lungs of A/J mice, but has a minor effect in B10.A mice. Characterization of macrophages and dendritic cells by flow cytometry in the lung infiltrating leucocytes (LIL) from anti-CD25-treated and untreated A/J and B10.A mice inoculated i.t. with 1×10^6^ *P. brasiliensis* yeast cells. At weeks 2 and 10 after infection lung cell suspensions were obtained and stained as described in Materials and Methods. The acquisition and analysis gates were restricted to macrophages. Macrophages (A), and dendritic cells (B) at weeks 2 and 10 of infection. The data represent the mean ± SEM of the results from 6–8 mice per group and are representative of two independent experiments. * (*P*<0.05), ** (*P*<0.01), and *** (*P*<0.001) compared with IgG treated controls or the susceptible strain.

By week 2 of infection, compared with IgG-inoculated controls, A/J depleted mice showed a higher number of F4/80^+^GR1^+^, F4/80^+^ IA^K+^ and CD11c^+^IA^K+^ myeloid cells. However, no differences between treated and untreated B10.A mice were found (Fig. 8A). Importantly, only in A/J mice depletion of Treg cells led to increased numbers of myeloid (CD11b^high^CD11c^high^) and plasmacytoid (CD11c^int^B220^+^) subsets of dendritic cells (Fig. 8B).

An opposed effect on phagocytes was seen at week 10 of infection. A diminished influx of almost all mononuclear phagocytes and dendritic cells were seen in the lungs of resistant mice. In B10.A depleted mice, only GR1^+^F4/80^+^ myeloid cells migrated in reduced numbers to the lungs (Fig. 8A,B).

### Treatment with Anti-CD25 Diminishes the Early Production of IL-10 and TGF-β but Increases the Levels of Th1/Th2 and Th17 Cytokines at the Late Phase of Infection

**Figure 9:**
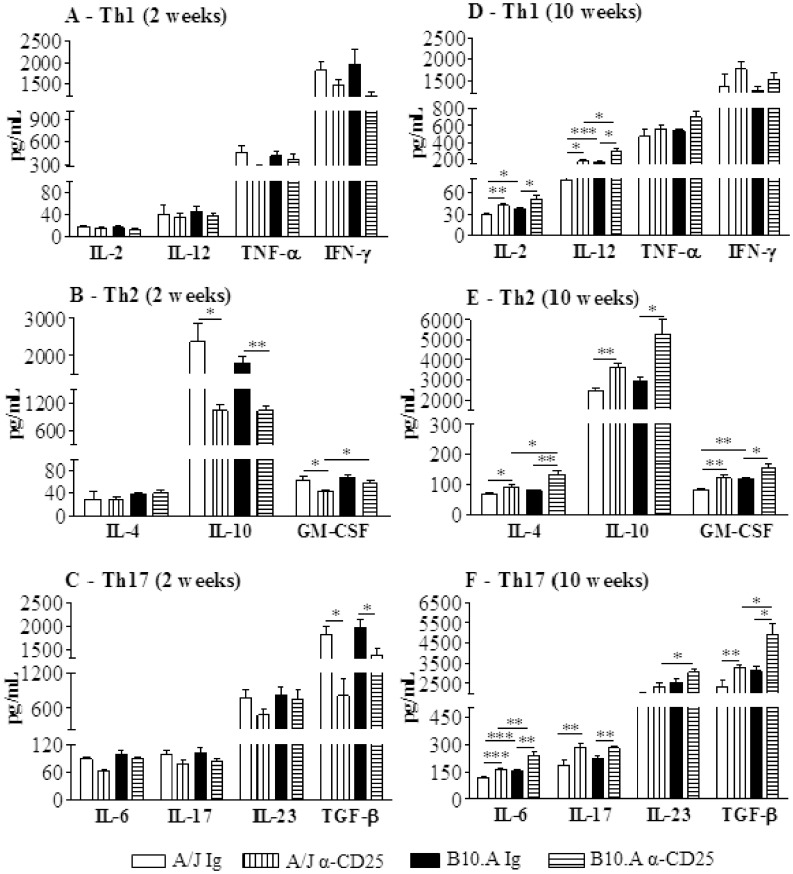
At week 2 after infection lungs from anti-CD25 treated mice presented decreased levels of IL-10, TGF-ß and GM-CSF, but at week 10 increased levels of Th1-, Th2-, and Th17-associated cytokines. At weeks 2 and 10 after i.t. infection with 1×10^6^ yeast cells of *P. brasiliensis*, lungs from anti-CD25 treated and untreated A/J and B10.A mice were collected, disrupted in 5.0 ml of PBS and supernatants analyzed for cytokines content by capture ELISA. (A, B, and C) Th1, Th2 and Th17 cytokines at week 2 of infection, respectively. (D, E, and F) Th1, Th2 and Th17 cytokines at week 10 of infection, respectively. The bars depict means ± SEM of cytokine levels (6–8 per group). The results are representative of two independent experiments. * (*P*<0.05), ** (*P*<0.01), and *** (*P*<0.001) compared with IgG treated controls or the susceptible strain.

The presence of cytokines was analyzed in lung homogenates obtained at weeks 2 and 10 after infection. Despite the reduced pulmonary fungal loads, no differences in Th1 cytokines were detected at week 2 of infection (Fig. 9A). However, besides GM-CSF, the inhibitory cytokines IL-10 and TGF-β appeared in lower levels in both mouse strains (Fig. 9B,C). In contrast with week 2, at week 10 almost all assayed cytokines appeared in increased levels in anti-CD25-treated mice. IL-2, IL-12, IL-4, IL-10, GM-CSF, IL-17, IL-6 and TGF-β were secreted in higher levels by anti-CD25-treated mice (Fig. 9D–F). Altogether, the cytokine data demonstrate that anti-CD25 treatment induces an early decrease of the inhibitory cytokines IL-10 and TGF-β concomitant with unaltered production of pro-inflammatory cytokines. At the late phase, the lower fungal loads were accompanied by increased levels of almost all Th1/Th2 and Th17 cytokines assayed.

### Depletion of CD25^+^ Cells Reduces the Pulmonary Inflammation in Resistant and Susceptible Mice

**Figure 10:**
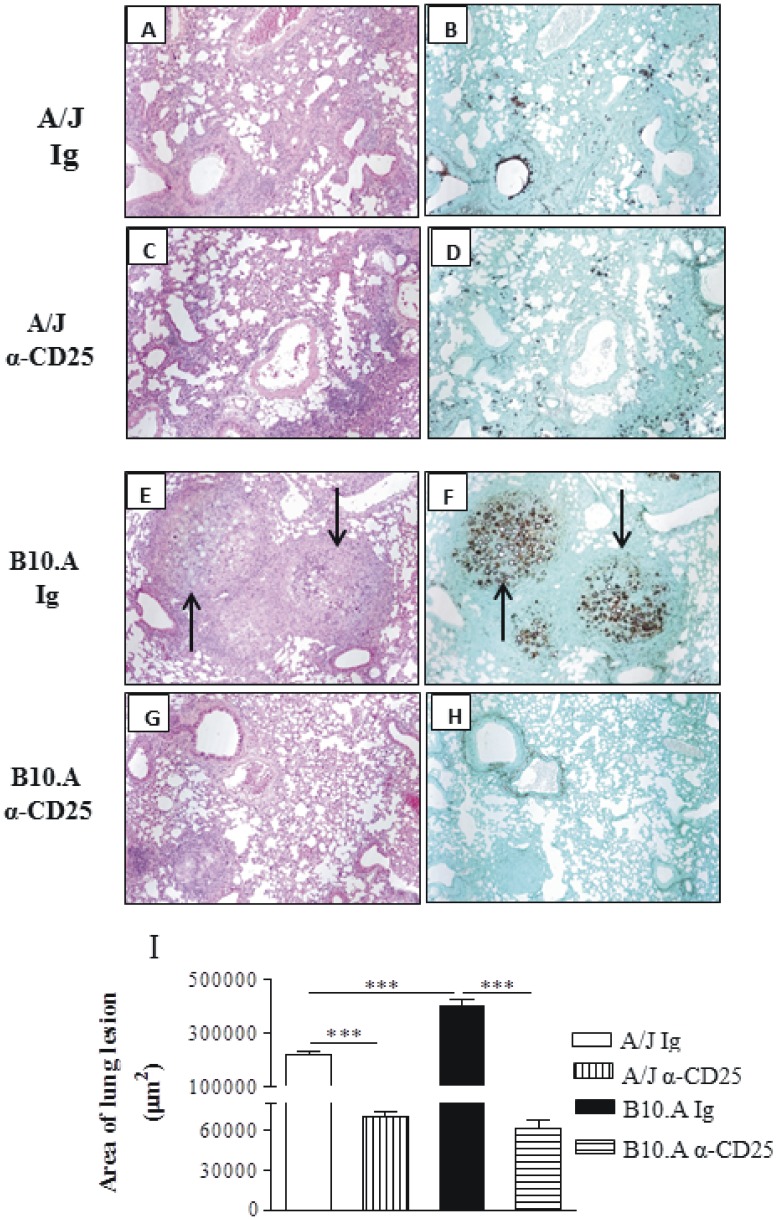
Histopathology of pulmonary lesions of anti-CD25-treated and untreated A/J and B10.A mice at week 10 post-infection with 1×10^6^ *P. brasiliensis* yeasts. IgG-treated (A, B) and anti-CD25-treated (C, D) A/J mice showed equivalent diffuse inflammatory reactions characterized by a small number of yeasts in the presence of elevated number of macrophages, lymphocytes and plasma cells; small portions of lung tissue were preserved, with limited signs of inflammatory cell recruitment. IgG-treated B10.A mice (E, F) presented an elevated number of well-defined, confluent, necrotic, granulomas of various sizes (E) containing an elevated number of fungal cells (arrows in F); these lesions occupy a large area of lung tissue (E, F). Compared with control B10.A mice, anti-CD25-treated B10.A mice showed significantly smaller lesions (G) containing a few number of yeasts (H). A, C, E, G, (HE, X 100); B, D, F, H (Groccot X 100). I- Total area of lung lesions of mice (n = 6) at week 10 after infection. ** (*P*<0.01), and *** (*P*<0.001) compared with IgG-treated controls or the susceptible strain.

The histopathology of lungs at week 10 of infection was very informative. IgG-treated A/J mice display a lymphomononuclear infiltration of lungs, preferentially localized in the interlobular septa close to the terminal bronchioles. Clumps of epithelioid macrophages organize as incipient granulomas, but well defined granulomas were absent. The number of fungi is small, and are sometimes absent from the lesions (Fig. 10A,B). Anti-CD25-treated A/J mice showed absence of granulomas and the lymphocytic inflammatory infiltrates are present in the interlobular and peribronchiolar septa, similarly to control A/J mice. The fungi are absent or scattered in the lung parenchyma (Fig. 10C,D). Control B10.A mice exhibit large granulomas involving extensive areas of lung parenchyma, containing large number of fungi (Fig. 10E,F). On the other hand, Treg depleted B10.A mice showed isolated incipient granulomas containing no fungus or a small amount of these cells. A more preserved area of parenchyma was also observed (Fig. 10G,H). The total area of lesions was quantified in histological sections and shown in Fig. 10I. At week 10, the lesion areas of B10.A control mice were significantly larger than those of control A/J mice. Importantly, depletion of CD25^+^ cells significantly diminished the areas of lesion in both mouse strains (Fig. 10I). In conclusion, the histopathological studies demonstrated that administration of anti-CD25 antibodies to resistant and susceptible mice results in decreased fungal burdens which evolves without deleterious enhanced tissue inflammation.

### Anti-CD25 Treatment Reduces the Number of Inflammatory T Cells and Macrophages in the Liver of A/J Mice but Only Decreases the Number of Macrophages in B10.A Mice

**Figure 11:**
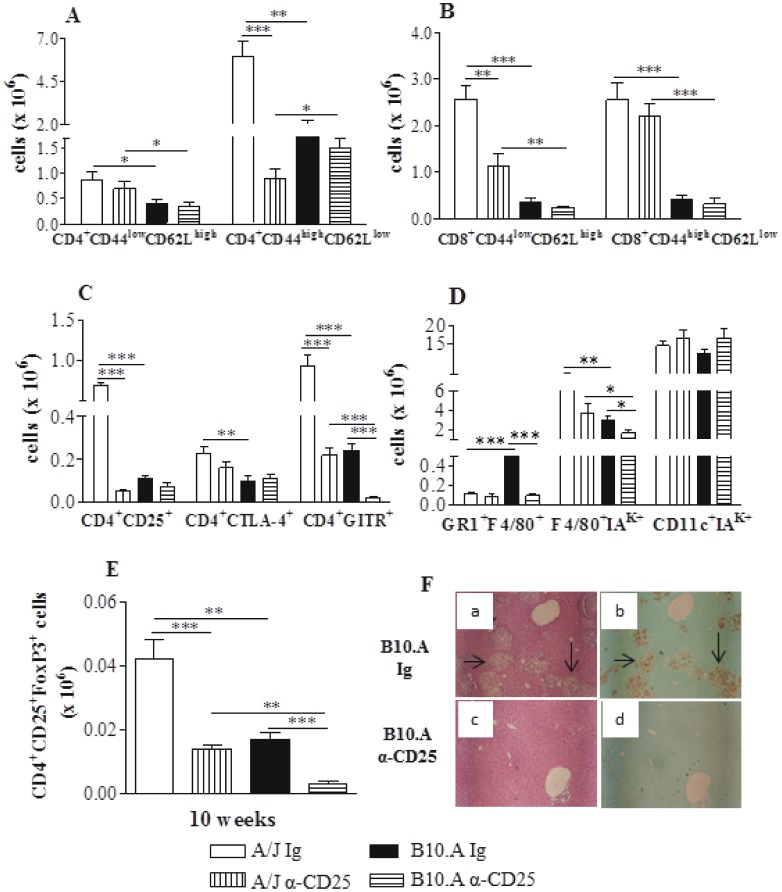
Depletion of CD25^+^ cells diminishes the inflammatory reactions in the liver of A/J mice and abolishes the hepatic fungal lesions of B10.A mice. Characterization of leukocyte subsets and activation profile of cells by flow cytometry in the liver infiltrating leucocytes (LIL) from anti-CD25-treated and untreated A/J and B10.A mice inoculated i.t. with 1×10^6^ *P. brasiliensis* yeast cells. At week 10 after infection liver cell suspensions were obtained and stained as described in Materials and Methods. The acquisition and analysis gates were restricted to macrophages or lymphocytes. A, CD4^+^ T cells; B, CD8^+^ T cells; C- Activated/Treg CD4^+^ T cells. D- Liver macrophages. E- CD4^+^CD25^+^Foxp3^+^ Treg cells. The data represent the mean ± SEM of the results from 5–6 mice per group and are representative of two experiments. * (*P*<0.05), ** (*P*<0.01), and *** (*P*<0.001) compared with IgG controls or B10.A strain. F- Histopathology of liver. Anti-CD25 treated and untreated A/J mice did not show hepatic lesions at week 10 of infection (data not shown). In contrast, control B10.A mice presented extensive hepatic lesions containing large numbers of fungal cells (F, a,b). Anti-CD25 treatment practically abolished the inflammatory lesions (F, c,d) and the fungal loads of B10.A mice. a, c (HE, X 100); b, d (Groccot X 100).

As dissemination and control of fungal growth in the liver appear to be an important marker of the regressive and progressive infections of A/J and B10.A mice [5], we studied the inflammatory infiltrates of this organ at week 10 of infection. Comparing control (IgG-treated) A/J and B10.A mice, it could be seen that resistant A/J mice had increased numbers of all T cell subsets analyzed, whereas in B10.A mice only the myeloid cells (GR1^+^F4/80^+^) predominated. Interestingly, Treg depletion in A/J mice led to diminished numbers of CD4^+^ and CD8^+^ (CD4^+^CD44^high^CD62L^low^, CD8^+^CD44^low^CD62L^high^, CD4^+^CD25^+^, CD4^+^GITR^+^) T cells (Fig. 11A–C). In B10.A mice, which presented a drastic reduction of CFU counts afteranti-CD25 treatment, only myeloid cells (GR1^+^F4/80^+^, F4/80^+^IA^k+^) appeared in decreased numbers (Fig. 11D). However, in both mouse strains a significant reduction in CD4^+^CD25^+^Foxp3^+^ Treg cells was found (Fig. 11E).

The histopathology of liver was also assessed in depleted and non-depleted mice.

At week 10 of infection, the small and equivalent numbers of viable yeasts present in the liver of control and Treg-depleted A/J mice were not sufficient to induce evident histological alterations. Thus, no inflammatory reaction and granulomas and absence of fungal cells were found (not shown). In contrast, granulomas localized in the lobules, containing a pronounced quantity of fungal cells were seen in control B10.A mice (Fig. 11F a,b). On the other hand, the livers of Treg-depleted B10.A mice had a normal aspect and did not present inflammatory reactions or fungal cells (Fig. 11F c,d).

### Anti-CD25 Treatment Abolishes the High Mortality of Susceptible Mice but does not Induce Sterile Immunity

**Figure 12:**
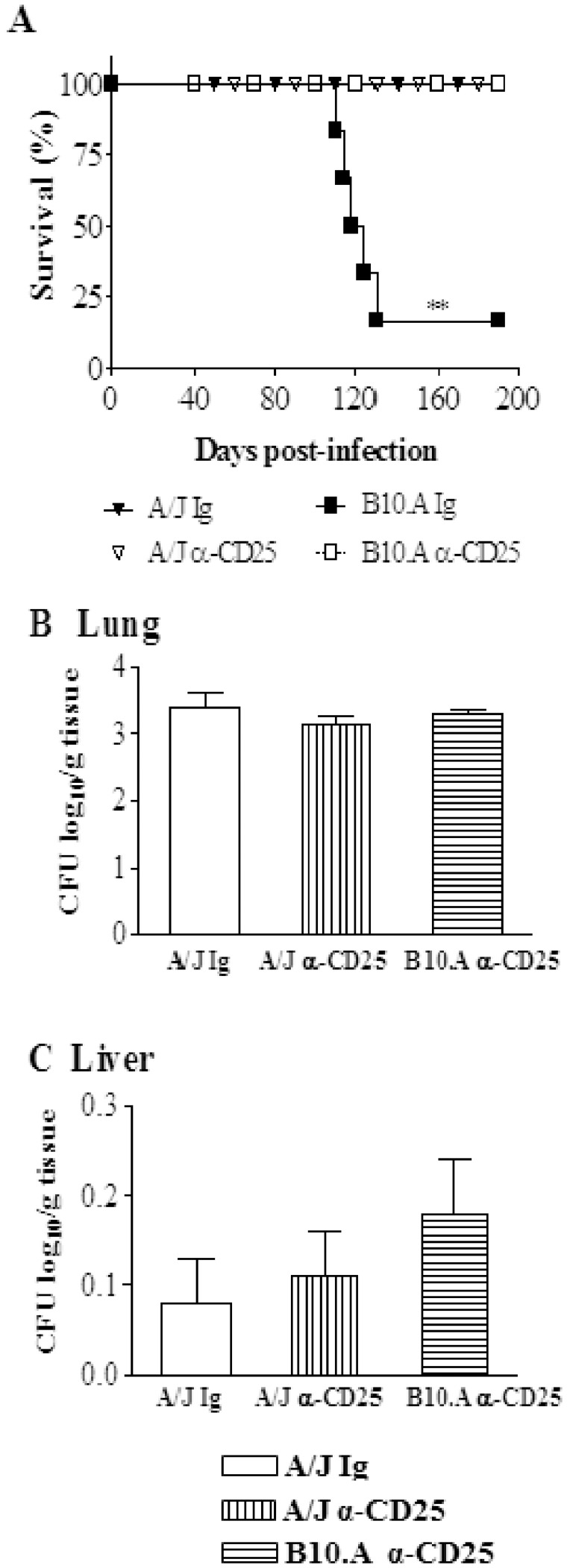
Depletion of CD25^+^ cells abolishes the increased mortality of susceptible mice but does not induce sterile immunity. A- Survival times of anti-CD25-treated and untreated B10.A and A/J mice (n = 6–7) after i.t. infection with 1×10^6^ *P. brasiliensis* yeast cells were determined in a period of 190 days. The results are representative of two independent experiments. **P<0.01. Recovery of fungal loads (CFU) from lungs (B), and liver (C) of survivor mice at day 190 after infection. The bars represent means ± SEM of log_10_ CFU obtained from groups of 6–7 mice. The results are representative of two experiments with equivalent results. **P<0.01.

To assess the influence of anti-CD25 treatment on the disease outcome, mortality of *P.brasiliensis* infected A/J and B10.A mice previously treated or not with anti-CD25 antibodies (n = 6–7) was registered daily after infection with 1×10^6^ yeast cells. As shown in Fig. 12A, at day 130 after infection 5/6 (83%) of control B10.A mice were dead. In the same period, all Treg-depleted B10.A and A/J mice, besides control A/J mice, were still alive.

To assess if the early depletion of CD25^+^ cells led to the sterile cure of mice, the presence of viable *P. brasiliensis* was analyzed in the organs of survivor mice at day 190 post-infection. As shown in Fig. 112B, low and equivalent numbers of viable yeast cells were recovered from the lungs of all studied groups. Furthermore, a very small number of yeast cells was still present in the livers (Fig. 12C) but no viable *P. brasiliensis* were detected in the spleens. Altogether, these data demonstrate that anti-CD25 treatment rescue susceptible mice from increased mortality caused by the progressive *P.brasiliensis* infection. In addition, this treatment did not induce a sterile cure of infection, but eliminated the differences in the disease outcome between susceptible and resistant mice to this fungal pathogen.

### IDO mRNA Expression, Kynurenine and NO Production are Reduced by Anti-CD25 Treatment of Infected Mice

**Figure 13:**
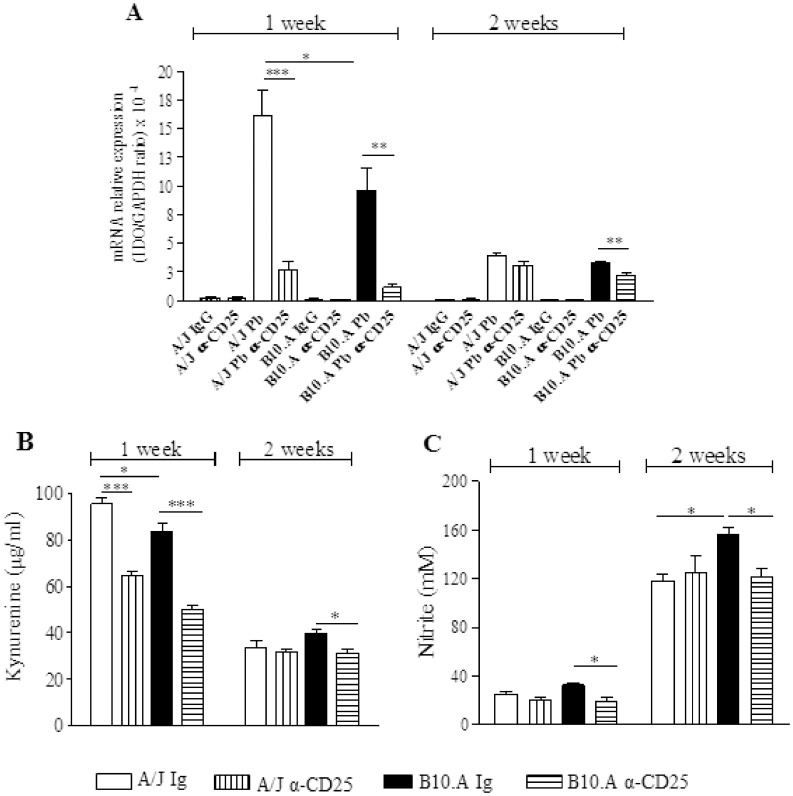
Anti-CD25 treatment induces reduced expression of IDO mRNA and diminished production of kynurenine and NO. IDO mRNA expression (A) in the lungs of IgG or anti-CD25 treated normal and infected B10.A and A/J mice was monitored by quantitative RT-PCR. The data are reported as a ratio of IDO/GAPDH. Lung homogenates were obtained from anti-CD25 treated and untreated B10.A and A/J mice infected with 1×10^6^ *P. brasiliensis* yeasts. The concentration of kynurenine (B) and nitrite (C) was measured in lung supernatants obtained at weeks 1 and 2 after infection. Concentrations of NO and kynurenine were measured using colorimetric assays. The bars represent means ± SEM of data obtained from groups of 6–7 mice. The results are representative of two independent experiments. * (*P*<0.05), ** (*P*<0.01), and *** (*P*<0.001) compared with IgG treated controls or the susceptible strain.

Indoleamine 2,3 dioxygenase is an enzyme involved in tryptophan catabolism. Its role in antimicrobial resistance by depleting tryptophan, essential for the growth of microorganisms, is well described [33], [47]–[50]. In addition, IDO has an important role in regulating T cell activation and preventing tissue pathology due to excessive immunological inflammation. Due to their capacity to induce Tregs and inhibit Th17, IDO and kynurenines contribute to define the subsets of T cells which are activated during fungal infections [47]–[50]. Our study demonstrated that anti-CD25 treatment depleted Treg cells and was associated with the activation of cytokines of the Th1/Th17 pathways. Thus we decided to investigate the role of anti-CD25 treatment on the expression of IDO and production of kynurenines. As shown in Fig. 13A, *P* *. brasiliensis* infection induced a high expression of IDO mRNA in the lungs of both, B10.A and A/J mice. This expression was higher at week 1 than week 2 of infection, and was higher in the lungs of A/J than in B10.A mice. In both mouse strains, anti-CD25 treatment led to decreased expression of IDO. Consistent with those findings, decreased levels of kynurenine were observed in the lungs of CD25-depleted mice (Fig. 13B).

Because NO has been described as the major anti-fungal compound in paracoccidioidomycosis [11], [51], we have also assessed the levels of nitrite in the lung homogenates of mice. Interestingly, an inverse correlation with kynurenines was found (Fig. 13C). At week 1 of infection, low levels of NO were concomitant with high levels of kynurenines; at week 2 an inverse picture was detected. Furthermore, by week 2 of infection, susceptible mice produced higher levels of NO than resistant mice and only in B10.A mice depletion of CD25^+^ cells resulted in decreased levels of pulmonary NO.

## Discussion

The murine model of paracoccidioidomycosis has some peculiarities that help to explain the results here observed. The resistance and susceptibility patterns of A/J and B10.A mice are manifested late in the course of *P.brasiliensis* infection. As reported before [5] and confirmed here, the initial infection is more severe in the resistant A/J mice, which allows for precocious and more intense dissemination of fungal cells than in B10.A mice. This behavior appears to be governed, at least in part, by innate immune cells. The alveolar macrophages and dendritic cells of A/J mice are permissive to fungal growth and synthesize large amounts of TGF-β, TNF-α and IL-6, followed by the late development of CD4^+^ and CD8^+^ effector T cells ([7], [52], Pina et al.submitted). During the chronic phase, cell-mediated immunity predominates and the infection is regressive in the lungs and in the dissemination organs. In contrast, the infection in B10.A mice is initially well controlled and characterized by early secretion of IL-12 and NO by alveolar macrophages and DCs ([5], [11], [52], Pina et al.submitted). Excessive production of NO, however, has been shown to cause T cell anergy, progressive infection and death in susceptible mice [9], [11].

The ex vivo analysis of Treg cells revealed some clues that help us understand the differential roles of Treg cells in resistant and susceptible mice. First, normal, uninfected A/J mice have higher baseline numbers of Treg cells in their lungs than B10.A mice. However, Foxp3^+^ Treg cells from both mouse strains express membrane LAP, CTLA4, GITR and intracellular IL-10 and TGF-β suggesting equivalent phenotypes and possibly mechanisms of action. During the course of infection, the numbers of Treg cells were always greater in the resistant mice. The elevated synthesis of TGF-β by alveolar macrophages and DCs of these mice ([52], Pina et al submitted), likely contributed to the greater number of Treg cells. The increased potency of A/J Tregs was concomitant with an elevated expression of Foxp3, CTLA4 and IDO which are markers of the suppressive ability of these cells [27]–[29]. Interestingly, in several other infectious processes, strong Treg responses also correlate with disease resistance. Resistance to persistent salmonellosis depends on the early development of highly suppressive Treg cells [28], and in invasive pneumococcal pneumonia, the early immune response in resistant mice is characterized by an increased synthesis of TGF-β and a rapid increase in the number of lung Treg cells [53]. However, in experimental leishmaniasis, higher suppressive activity of Treg cells was observed in susceptible BALB/c mice [54].

This study demonstrated that anti-CD25 treatment of resistant mice and susceptible mice resulted in persistent depletion of CD4^+^CD25^+^Foxp3^+^ Treg cells. At this point it can be argued that the use of anti-CD25 antibodies is not adequate to characterize Treg cells function, since this antibody also neutralizes and/or depletes other CD25-expressing cells such as activated/effector T cells. This is true and a matter of debate [44], [45], [55], but our data showed a significant decrease in the number of CD4^+^CD25^+^ Foxp3^+^ Treg cells that was sustained up to week 10 of infection. To further validate our observations, additional data using Foxp3^GFP^ C57BL/6 mice demonstrated that the anti-CD25 protocol here employed was able to significantly decrease the Foxp3^GFP^ Treg cell population, without affecting the expansion of activated T cells. Decreased fungal burdens and Treg cells (CD4^+^ Foxp3^GFP^), which were associated with increased numbers of effector CD4^+^ T (CD44^high^CD62^low^) cells, were detected at week 2 post-infection. Importantly, Treg cells were characterized by the expression of GFP and not by the use of anti-CD25 antibodies. Therefore, using a more specific system to identify Treg cells, we could observe a consistent decrease in Foxp3^GFP^ Treg cells in anti-CD25-treated mice associated with decreased fungal loads and increased effector T cells.

At week 2, the decreased fungal burdens of A/J mice were concomitant with an increased influx of naïve and effector CD4^+^ and CD8^+^ T cells to the lungs. Activated macrophages and dendritic cells also appeared in more elevated numbers, indicating a clear enhancement of T cell immunity. The partial depletion of CD4^+^CD25^+^Foxp3^+^ cells by anti-CD25 treatment could, at least in part, explain the low numbers of CD4^+^CD25^+^ cells detected in the lungs of the A/J mice at week 2 of infection. However, as activated/effector CD4^+^ and CD8^+^ T cells (defined by CD44 and CD62L expression) appeared in elevated numbers, it is possible that the remaining anti-CD25 antibodies were still blocking CD25 epitopes on T cells at week 2 of infection. At later phases, decreased numbers of all inflammatory cells were observed, indicating that the size of the fungal loads was controlling the migration of cells to the site of infection. Importantly, the decreased numbers of Treg cells did not cause the delayed exacerbated inflammatory reactions that are frequently detected in other fungal infections when Treg cells are ablated [31], [32], [56].

In the B10.A mice, no increased inflammatory reactions were observed despite the intense and persistent depletion of Treg cells following anti-CD25 treatment. At week 2, the diminished levels of IL-10 and TGF-β and the sustained number of inflammatory cells and pro-inflammatory cytokines in the lungs resulted in better control of fungal loads. At week 10, the enhanced production of TH1/Th2/Th3 cytokines associated with diminished numbers of inflammatory cells and fungal loads suggest the existence of more efficient effector mechanisms without concomitant tissue pathology caused by inflammatory reactions. Indeed, it has been described that in the presence of small numbers of Treg cells, fewer immune cells can more efficiently exert their effector activity [17], [56].

Fungal dissemination to the liver is an important marker of disease severity in B10.A mice. Liver inflammation in our model appeared to mirror our observations in the lungs at week 10 of infection. In both mouse strains, the lower fungal loads were associated with decreased numbers of Treg cells but decreased inflammatory reactions were detected only in resistant mice. The effects of anti-CD25 treatment on liver lesions in the susceptible mice were impressive. In contrast to the IgG-treated controls, no lesions or fungal cells were detected in the livers of the anti-CD25-treated mice, suggesting the development of highly efficient protective mechanisms. Thus, the great improvement in fungal clearance and the decreased lesion area in the lungs and liver appear to have rescued the susceptible mice from death. Furthermore, the anti-CD25 treatment did not lead to sterilizing immunity in the survivor groups (the control A/J mice and the anti-CD25 treated B10.A and A/J mice), suggesting that the remaining low numbers of yeast cells were able to induce prolonged T cell memory and control further fungal growth. As described in murine leishmaniasis and candidiasis, the persistence of pathogens conferred by Treg cells is important for maintaining memory cells and protective immunity that control further microbial challenges [30], [31].

The anti-inflammatory cytokine TGF-β drives the differentiation of naïve CD4^+^CD25^+^ T cells to CD4^+^CD25^+^ regulatory T cells by inducing Foxp3 [57]. In contrast, IL-6 prevents the induction of Foxp3 by TGF-β and instead directs T cells towards the Th17 lineage by inducing the RORγτ and RORα transcription factors [58], [59]. Our cytokine data at week 10 of infection demonstrated increased levels of Th17-associated cytokines without increased lung pathology. The increased levels of IL-10 also present in lung homogenates may have controlled the pathogenicity of IL-17-producing cells without inhibiting their protective function [59]. Moreover, anti-CD25 treatment of susceptible and resistant mice led to reduced numbers of Treg cells, IDO expression and kynurenines production. Thus, the modulatory activity of anti-CD25 antibodies on Treg cells appears to have potentiated the development of Th17 cells, possibly contributing to the control of fungal burdens. Indeed, our previous studies with C57BL/6 TLR2 deficient mice demonstrated that inflammatory PMNs induced by enhanced Th17 immunity are protective in paracoccidioidomycosis. However, the impaired production of IL-10 by TLR2-deficient mice allowed increased inflammatory reactions and lung pathology [38]. In another study, we demonstrated that in TLR4-deficient mice, reduced numbers of IL-17^+^ CD4^+^ T cells paralleled enhanced differentiation of Treg cells, demonstrating the opposing development of Treg and Th17 cells in paracoccoccidioidomycosis [39].

The data obtained in this study demonstrate that the number and activity of Treg cells, as well as the levels of IDO mRNA and kynurenines, are modulated by anti-CD25 treatment. Our previous results suggested that the immunoregulatory mechanisms of resistant mice are mainly governed by TGF-β, whereas pro-inflammatory mediators, such as those induced by the IL-12/IFN-γ axis, are prevalent in susceptible mice [9], [52]. In A/J mice, the elevated levels of TGF-β secreted by alveolar macrophages and DCs ([52], Pina et al. submitted), the high number of Treg cells and the increased IDO activity indicate the existence of an initial tolerogenic loop that is mediated by these cells and mediators. In fact, it has been shown that Treg cells utilize TGF-β to maintain the tolerogenic function of dendritic cells by inducing the ITIM (immunoreceptor tyrosine-based inhibitory motif) mediated signaling properties of IDO molecules. However, the IFN-γ-induced enzymatic activity of IDO leads to tryptophan starvation and the generation of kynurenines, which are immunoregulatory catabolites that promote the conversion of naïve T cells into Foxp3^+^ Treg cells [60]–[62]. During the early phase of the infection, the relative control of fungal growth in B10.A and A/J mice is partially IDO-mediated [Frank de Araújo, manuscript in preparation]. However, as was observed in this study, the enhanced migration of activated T cells and macrophages to the site of infection, which was observed only in resistant mice, was associated with anti-CD25 treatment and reduced numbers of Foxp3^+^ Treg cells. In other words, the early immunosuppression observed in A/J mice appears to be tightly regulated through a TGF-β-Treg-centered mechanism that utilizes IDO to clear fungal cells and induce tolerogenic dendritic cells. Moreover, anti-CD25 treatment appears to block this suppressive loop, such that T cells can expand and migrate to the site of infection.

Albeit at lower levels, B10.A mice also produced significant levels of IDO and kynurenines and developed elevated numbers of Treg cells after one week of infection. However, the reduced production of IDO and kynurenines induced by anti-CD25 treatment was not able to restore the impaired T cell migration to the lungs. Our previous studies showed that, in response to *P. brasiliensis* infection, the alveolar macrophages and DCs in B10.A mice secrete high levels of IL-12 and activate the secretion of IFN-γ by innate immune cells [7], [10], [52]. These pro-inflammatory mediators induce the excessive production of NO and IDO, which controls fungal growth and exerts a prominent role in the immunosuppression of susceptible mice [9], [11]. Consistent with these observations, the in vivo depletion of NO [11] and the inhibition of IDO activity by 1-methyl tryptophan [Frank de Araújo, manuscript in preparation] restored T cell immunity and the early migration of T cells and macrophages to the lungs of B10.A mice. Thus, the early immunity generated in susceptible mice appears to be governed by pro-inflammatory mediators (e.g., IL-12, IFN-γ and NO) that likely regulate the IDO-mediated control of Treg cells. In the present study, anti-CD25 treatment also led to diminished levels of IL-10 and TGF-β, which reinforced the suppressive pro-inflammatory milieu in the lungs of B10.A mice [9], [11]. However, this immunological balance that reduced fungal loads without causing excessive tissue damage was able to rescue susceptible mice from precocious death.

In conclusion, our studies are the first to demonstrate that Treg cells exert deleterious effects on the mild and severe forms of paracoccidioidomycosis. These results highlight new perspectives for understanding the immunopathology of this infection. For example, previous studies generally associated the presence of Treg cells with severe cases of paracoccidioidomycosis [35]–[39]. Importantly, our data demonstrate that anti-CD25 treatment causes a partial depletion of Treg cells and abolishes the differences between the severe and mild forms of paracoccidioidomycosis, which indicates that the manipulation of Treg cells may prove to be a new potential therapy against this important systemic mycosis.
